# New-type urbanization ecologically reshaping China

**DOI:** 10.1016/j.heliyon.2023.e12925

**Published:** 2023-01-25

**Authors:** Jiangyan Wang, Suwan Han, Han Lin, Pingping Wu, Jingfeng Yuan

**Affiliations:** a School of Statistics and Data Science, Nanjing Audit University, China; b School of Engineering Audit, Jiangsu Key Laboratory of Public Project Audit, Nanjing Audit University, China; c Department of Construction and Real Estate, Southeast University, China

**Keywords:** National new-type urbanization plan, Difference-in-difference, Green urban planning, Propensity score matching, PM_2.5_ concentration

## Abstract

Addressing critical ecological issues is one of the core objectives of the Chinese National New-type Urbanization Plan (NTU). However, there is not enough research to clearly demonstrate NTU's impact on environmental pollution. There is a need to provide solid empirical evidence from evaluation of the effects of NTU on environmental pollution. This study implements a series of propensity score matching and difference-in-difference analyses based on a comprehensive panel dataset spanning the period 2006–2017. The quantitative results show that NTU is largely conducive to curbing environmental pollution, with PM_2.5_ concentrations as a proxy, and its robustness is demonstrated by the parallel trend test and placebo test. Further, the ecological effect of NTU is heterogeneous in terms of city level, location, and former pollution level. Small and medium cities benefit more than larger or central cities from NTU, and eastern cities exhibit better air quality improvement than cities in western regions. In addition, through quantile regression, we find that the positive outcomes of NTU are restricted by level of pollution, i.e., heavier pollution adds greatly to the difficulty of abatement.

## 1 Introduction

China is pursuing a grand urban dream [[1], [2], [3], [4], [5], [6], [7]]. Between 1978 and 2019, China's urbanization rate increased from 17.92% to 60.60%. This, the world's largest and fastest urbanization process, not only significantly fuels China's dramatic economic growth, it also substantially changes the social structure [[8], [9], [10], [11], [12], [13]]. Traditional forms of urbanization bring about high consumption of resources and energy as well as deterioration of the ecological environment, which unavoidably impacts both the nation and other parts of the world [[14], [15], [16], [17], [18]]). The presence of persistent smog and high levels of fine particulates (PM_2.5_) have acted as a tipping point for China's clean air movement in recent years, which has been increasingly active in stimulating debates about these issues among the urban Chinese public, the government, academia, and the media [15,16].

To address sustainability challenges, the Central Committee of the Communist Party of China and the State Council of China jointly launched the National New-type Urbanization Plan (NTU), the first national strategy on urbanization, in March 2014. This comprehensive and ambitious plan aims to achieve four major tasks, including ecological progress, urbanization quality, expanding domestic demand, and rural–urban coordination. Beyond traditional issues, NTU pays great attention to human development and social welfare by emphasizing a sustainable and people-centered approach [1,[19], [20], [21], [22]]. Its core lies in sustainable and coordinated advancement of issues related to the economy, population, society, and the environment [23,24].

In fact, urbanization may be a double-edged sword [6]. Rapid urbanization has caused many eco-environmental problems, which also reduce the quality of urbanization and threaten the sustainable development of urban [25,26]. From the environmental perspective, the effort to reach NTU targets would depend heavily on a construction boom, including approximately 30 million additional units of housing and tremendous corresponding infrastructure [2,14]. Unfortunately, NTU cannot proceed without exacting a significant toll on the environment, because an uptick in consumption of water, energy, and land becomes inevitable.

Attaching a “green” label to new developments requires deep integration of ecology in the whole development process [6]. To fully realize the remarkable targets of the new blueprint, the National Development and Reform Commission officially announced the first batch of national comprehensive pilot areas, consisting of 62 NTU cities by the end of 2014. The second and third batches, comprising 59 and 111 cities, were issued in November 2015 and December 2016, respectively. As a coordinated, top-level effort to increase the population of China's cities, this national-scale scheme offers an enormous opportunity to introduce sustainable technologies and practices [1,2]. Tailored and adaptive policy approaches are conducted to trigger innovation and engagement with local contexts. For example, pilot areas are required to reinforce management and control of urban planning, speed up construction of green cities, and guarantee air pollution control actions. Green building standards are adopted to ensure that new construction meets benchmarks for sustainability. With regard to existing buildings, cost-effective and low-carbon retrofitting plans are implemented to increase energy efficiency. Greenbelts should be maintained to restrict undue urban sprawl and alleviate vehicle exhausts. By 2020, NTU had brought about globally significant changes to China, such as a rapidly increased GDP, a world-leading transportation system, and outstanding education and healthcare facilities [2,9,27,28]. Nevertheless, things are not as they appear. In fact, NTU gives higher priority to the environment than to economic development, and the coordinated development between urbanization and the environment is vital in the construction of ecological civilization [29]. In the last decade, clarifying the evolution laws between urbanization and ecological environment has become a hot topic and frontier [30]. Research on the relationship between urbanization and ecological environment is developed from perspectives such as land use [7,28,31], urban hydrology [32,33], air pollution [14,23,25,34,35], energy and environmental effectiveness [12,15,36,37], landfill system [38], carbon emissions [[39], [40]].

Thus, the question arises: Has NTU maintained the “eco” orientation implied in its name, and does it go beyond the traditional pattern, which is factor-dependent and investment-driven? In other words, has urbanization gone hand-in-hand with environmental improvement? Although the relationship between urbanization and environmental pollution has been studied, there remain several limitations regarding the historical backdrop [17,18]. For instance, previous studies of the association between urbanization and environment emphasis on the development of evaluation systems [6,28,31,41], or only concern in the context of traditional urbanization [18,38,[42], [43], [44]], not NTU. While even if it was conducted in the background of NTU, only one or several provinces of China were considered [7,8,24,25,29,33,35,36,41,45], therefore, a desirable quantitative analysis should be proceeded to assess the ecologically effect of NTU.

To answer our research question, we present empirical evidence for a significant ecological effect from NTU. Using a panel dataset from 226 prefecture-level cities over the period 2006–2017, our intriguing and robust results based on difference-in-difference (DID) and propensity score matching (PSM) show that NTU exhibits a clear ecological effect; that is, implementation of NTU generally helps to reduce environmental pollution. We also show that the ecological effect of NTU has contextual heterogeneities. This positive phenomenon tends to occur in small and medium cities and eastern regions. Heavier pollution often weakens improvements stemming from NTU. A parallel trend test and placebo test are conducted to guarantee the reliability of our findings. This study offers three main contributions in advancing the understanding of NTU's effects on the ecological environment. First, it empirically draws an outline of both direct and dynamic ecological effects from NTU. Evidence-based conclusions stamp a veritable brand on the new urbanization process, which is eco-friendly and sustainable. Implementation of NTU accelerates modern industrial layout and development of urban–rural integration; it also harbors a distinct agenda of facilitating a sustainable transition toward a people-centered orientation. Second, it offers a fine-grained picture of unbalanced NTU outcomes using prefecture-level panel data. High-level policy intervention would result in a variety of diverse consequences. Regional heterogeneity and path dependence should be taken into account to optimize spatial distribution and promote balanced development. Third, this study employs a DID-based research design to effectively identify the outcomes of policy deployment. While NTU is a typical natural experiment, our methods provide a feasible way to reveal causal relationships between NTU and pollution reduction.

## 2 Background

### 2.1 China's new-type urbanization

China is advancing urbanization at an unprecedented rate [1,18,24]. Urbanization is a complex process, and the ongoing progress and expansion of urban areas have not only fueled economic growth and social prosperity but also profoundly influenced health and sustainable development [46,47]. Factor-dependent and investment-driven urbanization simply pursues an increase in population and industrial agglomeration, and has led to several problems such as inefficient land use, unreasonable spatial distribution, and serious environmental damage [19,32]. This traditional urbanization model faces difficulties in maintaining economic growth while combating the rising trends of congestion, air pollution, and urban sprawl. In light of the historic chance to realize the dream of sustainable urbanization, a remarkable shift from a traditional land-centered approach to a people-centered one is gaining momentum [22,37]. In March 2014, the NTU was released as a national strategy to accelerate this approach by rectifying existing problems and fostering an ecology-centered civilization [3,23].

The NTU aims to engineer an elaborate system of coordinated urbanization to bring about a new and inspiring vision of human-centered advancement. It is ambitious, intricate, and challenging, covering four key schemes: ecological improvement, urbanization quality, enhancing domestic demand, and urban–rural coordination [7,46]. Given that urbanization is a dynamic, multidimensional socio-spatial process, great efforts should be made to achieve a series of NTU targets. Above all, it is indispensable that people are at the core of NTU [20]. Unfolding this comprehensive top-down campaign requires more responsible practices, including protecting individual rights, boosting revenue for farmers, providing adequate social security and services for migrants, and decoupling GDP growth and socioeconomic development [16,17]. The urbanization ratio is projected to rise to approximately 70% (approximately one billion people) by 2030. More than four thousand trillion yuan is required to fund NTU [20]. The urbanization rate and investment demands vary greatly in different regions of China. Thus, appropriate plans related to the economic base, industrial structure, population agglomeration, infrastructure, and public services should be worked out to ensure a better life in cities and surrounding regions. For example, the accelerated reform of the Hukou system and fiscal, tax, and social security systems could facilitate the smooth transfer of rural migrants to urban areas [19]. Broadened channels of public participation might guarantee the processes of planning and implementation [1]. Special care must be taken to preserve historical landmarks and local or ethnic characteristics during the redevelopment process. Similar to other parts of the world, China's urbanization progress is closely associated with modernization. From 2009 to 2019 in China, the contribution of agriculture to GDP has shrunk from 9.6% to 7.1%, along with a significant decrease in the agricultural workforce. More institutional and systemic transformations are needed to accelerate innovation, upgrade the industrial structure, unleash the potential of labor transfer, and seize the strategic opportunities opened by NTU [10,22,40].

### 2.2 NTU and the environment

With variations in demographic change, economic activity, land cover and traffic patterns, urbanization plays a significant role in relation to air quality, which is significant in developing regions [48]. China faces serious environmental problems, especially air pollution [13,14,35]. The dual pressures of air quality and climate change mitigation heavily restrain high-quality growth [23]. Any adjustment of economic structures and development patterns is expected to be oriented toward marrying development with sustainability. Rapid urbanization is inevitably linked to environmental problems [6,17,35,40]. First, rapid urbanization creates strong demand for transportation-related infrastructure construction. Grand engineering schemes increase consumption of water, coal, cement, steel, energy, and land and may exact a heavy toll on the environment [49,50]. From 2001 to 2015, emissions of industrial wastewater, gases, and materials increased at an average annual rate of 4.0%, 14.2%, and 11.4%, respectively [10]. Total emissions of major pollutants will remain at a high level in the future if urbanization follows the established path. Second, rapid urbanization brings about intensive population accumulation and industry aggregation, which will exacerbate environmental hazards. By neglecting contextual development, traditional urbanization single-mindedly emphasizes external expansion. A 1 percentage-point increase in urbanization level in the period 2000–2015 corresponded to increased cement consumption of 1170.43 million tons, 386.26 million tons of steel, and 2359.65 million tons of standard coal [10]. With respect to efficiency, a 2.96% annual growth rate in urbanization corresponded to a 9.59% annual growth rate in consumption of cement, 13.56% for steel, and 7.42% for energy. Energy consumption for per 10 000 Yuan GDP was more than twice the global average in this period. Third, rapid urbanization has substantially changed people's lifestyles. With improved living standards, urban residents consume more products and services, leading to more pollution, and eventually worsening the natural environment [1].

NTU naturally appears at the junction of the dual constraints of resources and the ecological environment. It aims to integrate ecological concepts and principles into the entire urbanization process to reduce pollution [6,17]. On the one hand, NTU gives priority to the environment and focuses on green development. It sets up a series of indicators to ensure the ultimate goal, which is to attach a green label to the transformation of urban areas. Eco-friendly measures are deployed to promote green, circular, and low-carbon development. For example, low-carbon infrastructure investments are adopted to foster climate mitigation [51]. Greener urban planning facilitates lower traffic congestion, promotes land use efficiency, ameliorates population distribution, and reduces pollution emissions [16]. Higher environmental standards, stricter regulation, and systematic preferential policies would propel actors to strengthen urban greening [21,24]. On the other hand, population and industry aggregation are a prelude to introduction of green technologies and practices on a national scale. Cities could adopt more centralized treatments to address pollution emissions [37]. Optimization and upgrading of industrial structures may also trigger green innovation, intensive production, and a circular economy [15,40]. Advancements in coordinating population, industry, space, resources, and society are decoupling environmental costs (e.g., PM_2.5_ concentrations) and socioeconomic development.

As a tipping point for China's clean air movement, high levels of PM_2.5_ have aroused people to realize the seriousness of environment problems. The authoritative position of environmental governance was further strengthened since the 18th National Congress. In 2013, the Air Pollution Prevention and Control Action Plan (2013–2017) and the Chinese new Air Quality Index (AQI) have been issued. By adding in PM_2.5_, O3 and CO, the AQI considers six pollutants (PM_10_, PM_2.5_, NO_2_, SO_2_, CO, and O_3_). In 2015, the Law of the People's Republic of China on the Prevention and Control of Air Pollution was amended, which is called the “strictest ever” law on the prevention and control of air pollution, and the “Opinions on Accelerating the Ecological Civilization Construction” was issued, which is the top-level design document to guide China to carry out the ecological civilization construction in an all-round way. The “Opinions” makes an overall plan for promoting the ecological civilization construction in China. For the first time, the concept of “greening” was put forward, which, along with the new industrialization, urbanization, and agricultural modernization, endowed the construction of ecological civilization with new connotation. The ecological civilization was eventually incorporated into the constitution in 2018.

Recent years have seen research on the impact of urbanization, such as economic growth, environmental pollution of China [38], it is found that environmental pollution has a significant inhibitory effect on urbanization, and that there is an environmental Kuznets inverted U curve between urbanization and environmental pollution. Wang et al. [29] identified some influencing factors of urbanization, economy and environment in Shandong province, and pointed out that the coordination degree between economy and urbanization subsystem was the lowest. While the study of the effect of launching NTU on the environment is relatively less, which is accompanied by China's rapid urbanization. Urban air pollution is one of the most visible environmental problems [43], so there is a long way to go to promote the construction of new green urbanization. That is why we are interested in investigating the relationship between NTU and ecological environment, instead of the comprehensive relationship of economic and environment. Therefore, it is crucial to measure how the Chinese government intends to implement NTU in the context of ecological civilization. While environmental protection has certainly become more important under the “new normal”, strengthening environmental governance is essential to achieving the goal of environmental sustainability [52].

However, the details of policy implementation are important. Powerful policy intervention, if it is to succeed, must be adaptive. A one-size-fits-all scheme might invite malpractice and conflict with local interests. All endeavors should deliberately be made in tandem with contextual constraints, conditions, and opportunities. In fact, top-down endowments may lead to an over-reliance on national design, rather than local characteristics. Lack of supervision, assessment, and third-party monitoring also relax constraints and stand in the way of achieving green targets. The pilot urban areas offer a golden opportunity to test outcomes from implementation of NTU. Comparing the differences in the treatment group (NTU pilot urban areas) and the control group (non-pilot urban areas) before and after implementation of NTU could well evaluate the effects of relevant policies. Therefore, adding to the literature, this study conducts a meticulous environmental assessment of NTU implementation, improving our understanding of the environmental changes stemming from current urbanization.

## 3 Data and summary statistics

We construct a longitudinal city–year dataset to test the ecological effect of NTU for 226 prefecture-level cities in China over the period 2006–2017. The data are collected from several sources. First, information on NTU pilot areas is obtained from the National Development and Reform Commission. Second, prefecture-level pollution data are derived from the Modern Era Retrospective analysis for Research and Applications Aerosol Reanalysis (MERRAero), based on the Goddard Earth Observing System Data Assimilation System version 5 (GEOS-5) model driven by MERRA meteorological reanalysis [53]. Third, climatic data and sociometric characteristics for each city are gleaned from the National Meteorological Information Center, China Statistics Yearbooks, and Chinese Research Data Services (CNRDS). Finally, the data selection process generates 2625 observations. Since three batches of NTU pilot areas were launched in 2014–2016, this allows our test of the change in environmental pollution in the two groups to be more convincing when adopting the DID approach.

### 3.1 Environmental pollution

Various methods can be used to reflect the levels of environmental pollution. For example, sulfur oxide and water footprints could be used to measure environmental sustainability performance [54] as well as urban carbon emissions, PM_2.5_, PM_10_, NO_2_, chemical oxygen demand (COD) [34,55]. Among which, PM 2.5 is deemed related to the level of NTU [35,56]. As a major atmospheric pollutant, PM_2.5_ is extremely harmful to human health. In this paper, we focus on the concentration of PM_2.5_, which is a widely-used environmental indicator. One reason for choosing PM_2.5_ is that it is the primary measure of air pollution adopted in China [42]. Another reason is that almost all key pollution sources produce and report PM_2.5_, whereas pollutant like SO_2_ are tightly controlled by large state-owned enterprises in certain areas of China, rather than by local governments at the county level [57]. In addition, our explanation concerns the policy effect of NTU exerting on environment, while most other pollutants have only partial-year statistics (e.g., nitrogen oxides), it is more feasible to opt for PM_2.5_, which are relatively complete [58]. Consequently, given the relevance of PM_2.5_ pollution in the Chinese government's environmental targets [35], prefecture-level yearly concentration of PM_2.5_ is taken as the dependent variable.

Following Buchard et al., [53], yearly concentration of PM_2.5_ is calculated as.

PM_2.5_ = [DUST_2.5_] + [SS_2.5_] + [BC] + 1.4 × [OC] + 1.375 × [SO_4_].where [DUST_2.5_], [SS_2.5_], [BC], [OC], are [SO_4_] are the concentrations of dust, sea-salt, black carbon, organic carbon, and sulfate particulates with diameter ≤2.5 μm.

**Fig. 1:**
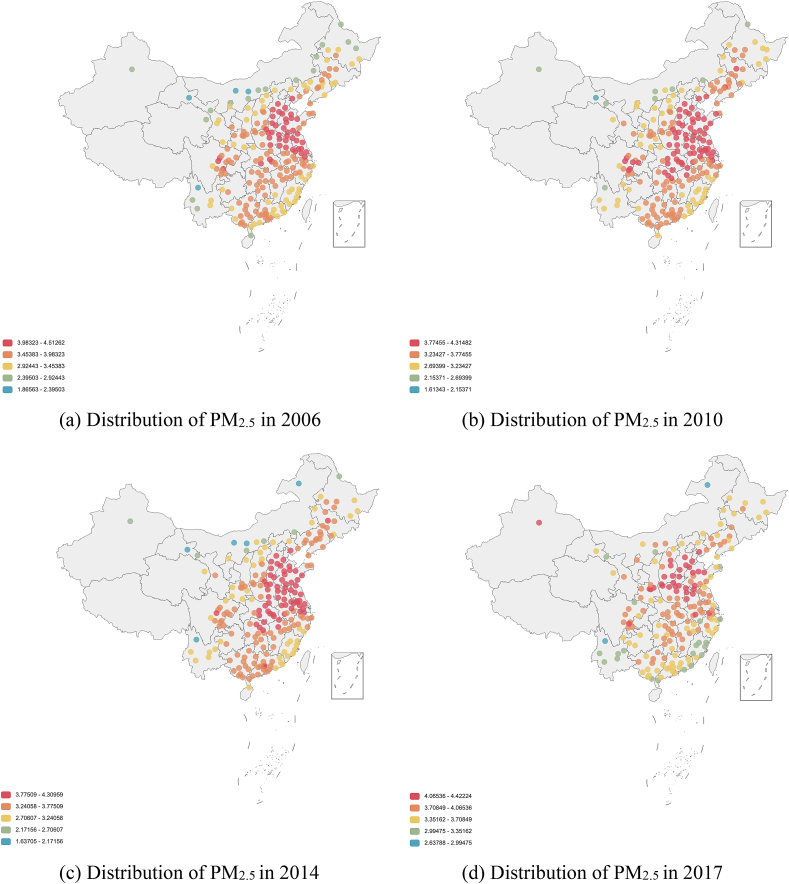
Spatial distribution of PM_2.5_ pollutants in China.

Distribution of PM_2.5_ is shown in Fig. 1, where the colors indicate degree of pollution in prefecture-level cities, with PM_2.5_ proportion in 2006, 2010, 2014, and 2017. Generally, one observes from Fig. 1 (a)–(d) that: (1) PM_2.5_ pollution is distributed heavily in the eastern region as well as Sichuan, which is located in the southwest; (2) PM_2.5_ pollution tends to concentrate in three super-urban agglomerations, probably caused by the development of heavy industries; (3) the northeast of China became significantly darker between 2010 and 2014, revealing that PM_2.5_ pollution became more serious, while the colors for the southeast and central regions are lighter.

### 3.2 Core explanatory variable

The core explanatory variable is taken to be event_*i*_ × time_*t*_, a dummy variable that takes the value 1 where the *i*-th city adopts NTU in the current and subsequent years, otherwise 0 [[59], [60], [61]]. In addition, the coefficient of event_*i*_ × time_*t*_ measures the net effect of environmental pollution.

### 3.3 Control variables

We group control variables that might influence the effect of NTU on environmental pollution as follows: 1) The level of economic development is measured by per capita GDP of each prefecture-level city (*Econ*). In fact, as an outcome variable of other socioeconomic variables, controlling per capita GDP may reduce the likelihood of omitting variables that simultaneously contribute to environmental pollution outcomes and NTU. 2) GDP share of the secondary sector is considered to represent the level of industrial structure (*InduStru*). This affects the level of environmental pollution [62]. 3) The proportion of local scientific enterprise expenditure (*Sci*) and the proportion of local education expenditure in fiscal expenditure (*Edu*) are used to represent social development [63]. 4) The number of new contracts signed by foreign investors (*Invest*) measures foreign direct investment. This is used to rule out the “pollution shelter” hypothesis that developed countries move more polluting industries to developing countries. 5) A series of weather conditions are also controlled, including temperature (*Temp*), wind (*Wind*), and relative humidity (*Hum*).

Table 1 lists descriptions of the above variables, which are scaled by taking their logarithm.

## 4 Ecological effects of NTU

### 4.1 Model specification

This study employs implementation of NTU as a natural experiment with which to examine the role of a new development strategy as a determinant of regional environmental improvements. To address endogenous challenges, we exploit the starting time of each NTU pilot area across different cities in China. We adopt a DID strategy to estimate the influence of NTU by comparing the changes in yearly concentration of PM_2.5_ of cities that embrace NTU relative to those that do not. Accordingly, the specific DID model is:

(1) $$y_{it}=\alpha +\beta \;{event}_{i}\times {time}_{t}+\delta_{i}+\mu_{t}+\gamma X_{it}+\varepsilon_{it}\text{,}$$

where *y*_*it*_ represents the pollution level of a city, *δ*_*i*_ and *μ*_*t*_ denote the city fixed effect and year fixed effect, respectively, *ε*_*it*_ is a stochastic error item, event_*i*_ × time_*t*_ is a dummy variable for the *i*-th city adopting NTU at year *t*, and *X* represents the control variables. The coefficient *β* of Equation (1) is the DID estimator demonstrating the NTU net effect on pollution level, which is a main explanatory variable. If *β* < 0 and significant, NTU exerts positive effects on environmental improvements; if *β* is negative and significant, NTU restrains environmental improvements; if *β* is non-significant, NTU does not exert a significant effect on environmental improvements.

### 4.2 Identifying assumptions

Compared to studies with a single shock, the selected model avoids possible omitted variables coinciding with a shock that may directly influence environmental enhancements [30]. First, the DID approach assimilates time-invariant and unobserved variances between the treatment group (NTU cities) and the control group (non-NTU cities). Second, the research design rules out omitted time trends that are simultaneously associated with the implementation of NTU and regional conditions. Third, this setting offers an additional advantage as entry into NTU at exogenously distinct times in different cities represents multiple shocks to place-based urbanization policies.

Because selection of NTU pilot cities is determined by central provincial governments, we cannot assume that this choice is random. There is a possibility that the central government's NTU decisions are dependent on time-invariant characteristics such as regional economic conditions, environmental status, and political importance. Due to the above-mentioned advantages of the DID setting, this possibility does not threaten our identification.

In addition, endogeneity can be alleviated by combining DID with matching [57]. For each NTU pilot city in our data, we match it with the non-NTU city that has the most similar level of environmental status. Then, the DID estimators are applied to the matched sample. Our results are similar using both matched and unmatched samples. Conducting the matching before DID has the advantage that the test results for the parallel trends assumption are improved using the matched sample, because standard errors are reduced.

We also test the parallel trend assumption via an event study. In the test, we generate the lead- and lag-year indicators for the NTU implementation as independent variables and test whether the coefficients of the leads are statistically significantly different from zero. The results described below fail to reject the hypothesis that the NTU and non-NTU cities follow similar trends (see Table 3).

### 4.3 Baseline regression

The corresponding results of DID with a two-way fixed effect model are summarized in Table 2. Models A and B reflect the net effect of NTU on environmental pollution without the control variables for the year fixed effect and two-way fixed effect, respectively. According to these two models, the coefficients of NTU on environmental pollution are 0.06 and −0.084, significant at the 1% level, indicating that NTU does reduce environmental pollution for the two-way fixed effect. Models C and D add control variables and the fixed effects, and we observe that NTU has a remarkably positive effect on regional environmental pollution, with coefficients of −0.07 and −0.056, respectively, significant at the 1% and 5% levels. The estimated coefficients are negative and stable in all four models. This indicates that implementation of NTU by and large is conducive to curbing PM_2.5_ concentrations. That is, NTU well addresses critical ecological issues and improves urban ecology in general.

**Table 1:** The descriptive statistics of the main variables.

Variable name	Indicators	Observation number (Obs.)	Mean	Standard deviation	Min	Max
PM_2.5_	PM_2.5_	2625	3.5556	0.4661	1.0508	4.5357
GDP share of the secondary sector	InduStru	2625	3.8556	0.2218	2.6283	4.4502
GDP per capita	Econ	2625	7.6401	1.9219	4.1891	14.5811
the proportion of local scientific enterprise expenditure in fiscal expenditure	Sci	2625	−4.6615	0.8634	−8.2141	−1.5758
the proportion of local education expenditure in fiscal expenditure	Edu	2625	−1.7056	0.2584	−4.2581	−0.9744
the number of new contracts signed by foreign investors	Invest	2625	4.9100	0.5058	0.9549	7.9950
temperature	Temp	2625	3.1995	0.6321	1.9751	7.9352
wind	Wind	2625	4.2613	0.2025	3.5906	6.4336
relative humidity	Hum	2625	3.0991	1.6272	0.0000	8.5362

**Table 2:** Impact of NTU on environmental pollution.

Variables	Models			
	(A)	(B)	(C)	(D)
event×time	0.06*** (0.022)	−0.084*** (0.026)	−0.070*** (0.024)	−0.056** (0.025)
InduStru			0.038*** (0.005)	−0.040 (0.025)
Econ			−0.392*** (0.076)	−0.251*** (0.067)
Sci			−0.030*** (0.007)	−0.033*** (0.011)
Edu			−0.084*** (0.023)	−0.083*** (0.024)
Invest			−0.004 (0.007)	−0.010 (0.007)
Temp			0.141*** (0.037)	−0.083** (0.033)
Wind			−0.013 (0.008)	−0.004 (0.007)
Hum			−0.011 (0.027)	0.037 (0.025)
city fixed effects	YES	YES	YES	YES
year fixed effects	NO	YES	NO	YES
N	2625	2625	2625	2625
*R*^*2*^	0.005	0.282	0.235	0.327

***p < 0.01, **p < 0.05, *p < 0.1.

Additionally, the variance inflation factor (VIF) test is conducted to exclude multicollinearity issues. The VIFs for all variables range from 1.08 to 1.47, with a mean value of 1.25, indicating no severe multicollinearity.

### 4.4 Parallel trend tests

To ensure unbiased DID results, a parallel trend test is conducted. The measurement model is:

(2) $$Y_{it}=a+\sum_{k=-6}^{2}\beta_{k}E_{it}^{k}+X_{it}\gamma +\delta_{i}+\mu_{t}+\varepsilon_{it}$$

where *i* and *t* represent the index of city and year, respectively, $E_{it}^{k}$ is a dummy variable taking the value 1 if the *i*-*th* city adopts NTU at year *t*, and *k* is an index for the parallel test. In our setting, NTU is implemented in 2014, for which we denote *k* = 0 in Equation (2).

The results of the DID regression are summarized in Table 3. This table shows that the results for the years before implementing NTU are not significant at the 5% significance level. The results for the current year of NTU implementation are also not significant, while the results become negatively significant in the next two years, indicating a lagged effect from implementing NTU.

**Table 3:** Numerical results of the parallel trend test.

	Coefficients	Standard deviation	t	P>|t|
Before 6	0.026	0.016	1.650	0.100
Before 5	0.029	0.018	1.600	0.110
Before 4	0.020	0.014	1.400	0.162
Before 3	−0.014	0.014	−0.990	0.322
Before 2	0.002	0.017	0.120	0.905
Before 1	−0.046	0.025	−1.840	0.067
Current	−0.004	0.039	−0.110	0.914
After l	−0.314	0.054	−5.810	0.000
After 2	−0.286	0.058	−4.960	0.000
InduStru	−0.033	0.025	−1.340	0.182
Econ	−0.244	0.067	−3.650	0.000
Sci	−0.034	0.012	−2.790	0.006
Edu	−0.081	0.023	−3.490	0.001
Invest	−0.010	0.007	−1.420	0.157
Temp	0.082	0.033	2.500	0.013
Wind	−0.005	0.007	−0.760	0.447
Hum	0.022	0.024	0.910	0.363

**Fig. 2:**
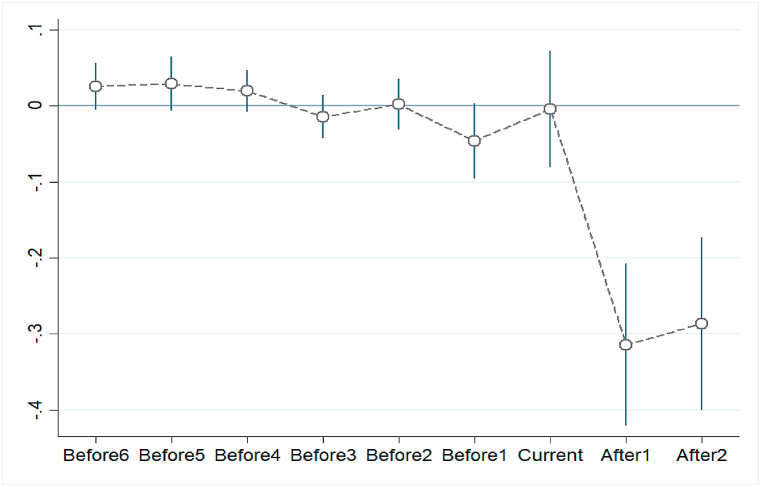
Plot of the parallel trend test.

The estimated coefficients of the lead- and lag-dummies are plotted in Fig. 2. This figure shows that the coefficient of the interactive term falls into the 95% confidence interval (solid vertical lines) before and in the current year of NTU implementation; that is, there are no statistical differences in PM_2.5_ pollution among the cities before implementing NTU, while the concentration of PM_2.5_ decreases 31.4% and 28.6% in the following two years after implementing NTU. This shows that, before implementation of NTU, the NTU and non-NTU cities had similar environmental pollution trends.

The parallel trend test also suggests that the timing of NTU implementation is unlikely to be endogenous to city environmental pollution, as the coefficients of the leads and lags are statistically insignificant in the first year before or after the year of NTU implementation. In the following two years, we also see that the lag term becomes negative and statistically significant.

To better capture the patterns of NTU treatment effects by year, a dynamic regression is explored starting from the year of NTU implementation. Estimated results are reported in Table 4. The first and second columns of this table show the results of the two-way fixed effect without and with control variables, respectively. We find that without the addition of control variables, the effect is positive and significant in the year of NTU implementation, while starting at the second year, the influence becomes negatively significant; similar performances are observed in the regression results after adding control variables. Hence, implementing NTU exerts a positive effect on environmental pollution, but it does so with a time lag of 1 year. Since the investigated cities have been enjoying NTU only for a short time, it is currently not possible to estimate the long-run effects of NTU on environmental pollution.

**Table 4:** Dynamic effect test.

Cases	Models	
	(B)	(D)
Current	0.054* (0.031)	0.075** (0.030)
After 1	−0.248 (***) (0.046)	−0.215 (***) (0.045)
After2	−0.229 (***) (0.051)	−0.187 (***) (0.048)
InduStru		−0.033 (0.025)
Econ		−0.248 (***) (0.067)
Sci		−0.031 (***) (0.012)
Edu		−0.084 (***) (0.023)
Invest		−0.009 (0.007)
Temp		0.078 (**) (0.032)
Wind		−0.005 (0.007)
Hum		0.026 (0.024)
city fixed effects	YES	YES
year fixed effects	YES	YES
*R*^*2*^	0.318	0.357

***p < 0.01, **p < 0.05, *p < 0.1.

### 4.5 PSM

Though the treatment assigned by randomization is a requirement of DID, cities launching NTU have different environmental conditions that are not randomly assigned. Thus, before performing DID, a sample with similar characteristics to the treatment group should be selected as the control group, to avoid selection bias. Under non-randomized treatment conditions, a matching resampling method, PSM is performed. PSM aims to construct counterfactual results by selecting non-NTU cities with conditions similar to the NTU cities. Consequently, the treatment and control groups show a common trend prior to NTU implementation.

**Fig. 3:**
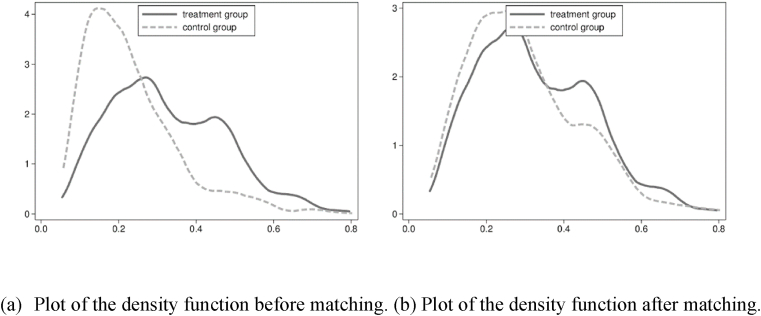
Kernel density plot of the control group (dotted line) and the treatment group (solid line) with (a): before matching, and (b): after matching.

Following Rosenbaum and Rubin [64], a balance test is carried out to evaluate the matching quality of propensity scores before PSM. Generally, the *t*-value between the matched treatment group and the control group should not be significant, and the standard deviation is valid below 20% in the balance test. A smaller standard deviation indicates higher matching quality. Table 5 shows the test results of the matching balance of propensity scores; after matching, the absolute values of deviations for all control variables are below 13%, dropping significantly, revealing that there is no significant difference between the two groups. To show this visually, a kernel density plot is depicted in Fig. 3, where the dotted line and the solid line represent the density of the control group and the treatment group, respectively. The two lines are close to each other after matching (Fig. 3b) compared with those before matching (Fig. 3a), which means that the common support set is relatively large.

**Table 5:** The balance test.

variables	matching status	Mean				*t*-test	
		treatment group	control group	Deviation rate (%)	Deviation reduction ratio (%)	t	*P*
InduStru	before matching	7.97	7.54	22.10	92.90	5.03	0
	after matching	7.97	7.94	1.60		0.27	0.76
Econ	before matching	3.86	3.85	4.30	−202.40	0.89	0.37
	after matching	3.86	3.84	12.90		2.35	0.02
Sci	before matching	−4.30	−4.77	54.60	87.20	12.65	0
	after matching	−4.30	−4.36	7.00		1.27	0.20
Edu	before matching	−1.72	−1.70	−7.70	−39.00	−1.68	0.09
	after matching	−1.72	−1.70	−10.70		−1.94	0.05
Temp	before matching	4.97	4.89	15.80	59.30	3.41	0
	after matching	4.97	5.05	−6.40		−1.19	0.23
Wind	before matching	3.19	3.20	−0.70	−339.50	−0.15	0.88
	after matching	3.19	3.18	3.20		0.60	0.54
Hum	before matching	4.30	4.25	29.30	69.00	6.02	0
	after matching	4.30	4.32	−9.10		−1.57	0.12
Invest	before matching	3.78	2.87	57.90	94.20	13.09	0
	after matching	3.78	3.85	−3.30		−0.59	0.55

Table 6 reports numerical results after PSM–DID analysis. Models A and B reflect the net effect of NTU on environmental pollution without the control variables, while models C and D add control variables. Note that after matching, the coefficients of the core variable *event × time* are significantly negative in all models. The corresponding coefficients of Models A and B are 0.059 and −0.698, respectively, significant at the 1% level. The coefficients of Models C and D are −0.084 and −0.055, respectively, significant at the 5% level, indicating that NTU may effectively reduce regional environmental pollution. The results of PSM–DID estimation are not significantly different from those of the previous DID regression, which further enhances robustness.

**Table 6:** The effect of implementing NTU after matching.

PM_2.5_	Models			
	(A)	(B)	(C)	(D)
event×time	0.059*** (0.021)	−0.084*** (0.025)	−0.698*** (0.023)	−0.055** (0.024)
InduStru			0.038*** (0.005)	−0.041 (0.026)
Econ			−0.394*** (0.077)	−0.252*** (0.068)
Sci			−0.030*** (0.007)	−0.034*** (0.011)
Edu			−0.086*** (0.023)	−0.083*** (0.024)
Invest			−0.004 (0.007)	−0.010 (0.007)
Temp			0.140*** (0.037)	−0.083** (0.033)
Wind			−0.014 (0.008)	−0.004 (0.007)
Hum			−0.013 (0.030)	0.035 (0.028)
city fixed effects	YES	YES	YES	YES
year fixed effects	NO	YES	NO	YES
*N*	2612	2612	2612	2612
*R*^*2*^	0.002	0.236	0.282	0.327

***p < 0.01, **p < 0.05, *p < 0.1.

### 4.6 Placebo test

To test the robustness of the DID framework and the estimates, we perform two placebo tests. A placebo model provides a procedure to investigate a model's reliability, since it basically introduces a placebo treatment that does not exactly correspond to the actual treatment used in the original model. We include in the placebo group that NTU was implemented before 2014; apart from this, the treatment and control groups are based on the same policy implementation. Since there was no NTU implemented during this period of time, the treatment group is chosen randomly. To verify the robustness of the proposed initial framework, no significant NTU impact should be measured, as the aforementioned placebo treatments are chosen arbitrarily.

### 4.7 Placebo test 1

Suppose that NTU was implemented before 2014. Two representative treatment years are set at 2009 and 2013. Table 7 reports the results of DID analysis supposing that NTU was implemented in 2013. Similarly, Models A, B, C, and D demonstrate DID results without and with control variables. We observe that all coefficients of NTU treatment are insignificant, except model A. Table 8 further reports two-way fixed effect DID results supposing that NTU was implemented in 2009 and 2013. Overall, the tested model settings consistently find no significant impact from the placebo setting. These findings support application of the DID framework.

**Table 7:** Change the year of implementing NTU to 2013.

PM_2.5_	Models			
	(A)	(B)	(C)	(D)
event×time	0.037*** (0.012)	−0.021 (0.015)	−0.221 (0.014)	−0.005 (0.015)
InduStru			0.035*** (0.005)	−0.043* (0.025)
Econ			−0.383*** (0.076)	−0.251*** (0.068)
Sci			−0.032*** (0.007)	−0.038*** (0.011)
Edu			−0.080*** (0.023)	−0.083*** (0.024)
Invest			−0.004 (0.007)	−0.010 (0.007)
Temp			0.145*** (0.037)	0.091*** (0.033)
Wind			−0.014* (0.008)	−0.005 (0.007)
Hum			0.007 (0.027)	0.031 (0.025)
city fixed effects	YES	YES	YES	YES
year fixed effects	NO	YES	NO	YES
*N*	2625	2625	2625	2625
*R*^*2*^	0.002	0.277	0.230	0.323

***p < 0.01, **p < 0.05, *p < 0.1.

**Table 8:** Numerical results of DID by assuming that NTU was implemented in 2013 and 2009.

PM_2.5_	Year 2013	Year 2009
event×time	−0.005 (0.015)	−0.019 (0.017)
InduStru	−0.043* (0.025)	−0.042 (0.025)
Econ	−0.251 (0.068)	−0.251*** (0.068)
Sci	−0.038*** (0.011)	−0.036 (0.011)
Edu	−0.083 (0.024)	−0.083*** (0.024)
Invest	−0.010 (0.007)	−0.010 (0.007)
Temp	0.091 (0.033)	0.091*** (0.033)
Wind	−0.005 (0.007)	−0.004 (0.007)
Hum	0.031 (0.025)	0.031 (0.025)
city fixed effects	YES	YES
year fixed effects	YES	YES
*N*	2625	2625
*R*^*2*^	0.323	0.324

***p < 0.01, **p < 0.05, *p < 0.1.

### 4.8 Placebo test 2

**Fig. 4:**
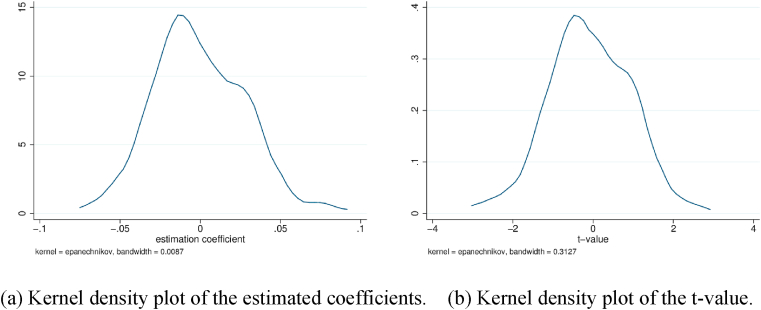
Kernel density plot of the estimated coeﬃcients (a) and t-value (b) over the 200 randomly generated treatment groups.

We randomly select 58 prefecture-level cities, where 47 cities are treated as the pseudo treatment group for 2014, 11 cities are treated as the pseudo treatment group for 2016, and the remainder serve as the control group. The above procedure is repeated over 200 times. The estimated coefficients and *t*-value kernel density plot of the randomly selected treatment groups are depicted in Fig. 4(a) and (b), respectively. The results show that the mean value of the regression coefficient is close to 0, and *t*-values are quite small. Table 9 presents the numerical results of 3 replicates using two-way fixed effect DID. Note that all core coefficients are insignificant. Therefore, the arbitrarily chosen NTU treatment group shows no explanatory power on variation in the environmental effects. Consequently, the placebo model settings underline the reliability of the statistical evidence obtained.

**Table 9:** Placebo test 2: randomly select the treatment group.

variables	replicate 1		replicate 2		replicate 3	
event×time	0.005 (0.032)	0.012 (0.030)	−0.026 (0.030)	−0.018 (0.027)	0.018 (0.032)	0.018 (0.030)
InduStru		−0.043* (0.025)		−0.042 (0.025)		−0.043* (0.026)
Econ		−0.251*** (0.069)		−0.251*** (0.068)		−0.250*** (0.068)
Sci		−0.038*** (0.011)		−0.038*** (0.011)		−0.038*** (0.011)
Edu		−0.083 *** (0.024)		−0.082*** (0.024)		−0.082*** (0.024)
Invest		0.007 (0.025)		−0.011 (0.007)		−0.010 (0.007)
Temp		0.092*** (0.033)		0.092*** (0.033)		0.092*** (0.033)
Wind		−0.005 (0.007)		−0.005 (0.007)		−0.004 (0.007)
Hum		0.030 (0.025)		0.030 (0.025)		0.030 (0.025)
city fixed effects	YES	YES	YES	YES	YES	YES
year fixed effects	YES	YES	YES	YES	YES	YES
*N*	2625	2625	2625	2625	2625	2625
*R*^*2*^	0.274	0.324	0.274	0.320	0.275	0.340

***p < 0.01, **p < 0.05, *p < 0.1.

### 4.9 Heterogeneity analysis

This subsection explores heterogeneity in the relative influence of NTU on regional environments in terms of city level, location, and former pollution level.

*Heterogeneity test 1*: Core and peripheral cities. To identify which type of city benefits more from NTU, we divide the sample cities into two groups: core and peripheral cities. Core cities are provincial capitals and municipalities. The estimation results are shown in Table 10. Columns 1–3 report the effects of NTU on peripheral cities and Columns 4–6 demonstrate the outcomes in core cities. Note that the estimated coefficients of the core variable are negative in all models, but significant only in peripheral cities. NTU implementation exerts a heterogeneous influence on diverse cities. Municipalities and provincial capitals are highly urbanized. They have natural advantages in scale, location, population, and resources due to their higher political status. They also own mature infrastructure and communication channels and have preferential access to fiscal subsidies and privilege policies. Moreover, NTU is not the task of the government, but the whole society, including various stakeholders [2]. Delivery of new urban development would be heavily depended upon by the main stakeholders. Peripheral cities find it easier to amend routine planning and implementation approaches due to their small scale and relatively undeveloped foundation. Thus, path dependence brings about lower adjustment and adaptation costs for peripheral cities than for core cities. NTU implementation can lever environmental improvements more effectively in peripheral cities.

**Table 10:** Heterogeneity test on the perspective of city-level.

Variables	Peripheral cities			Core cities		
event×time	−0.086*** (0.027)	−0.071*** (0.025)	−0.059** (0.026)	−0.055 (0.081)	−0.863 (0.070)	−0.033 (0.068)
InduStru		−0.040*** (0.005)	−0.038*** (0.025)		0.045*** (0.006)	−0.038 (0.028)
Econ		−0.395*** (0.077)	−0.253*** (0.068)		−0.418*** (0.090)	−0.274*** (0.080)
Sci		−0.030*** (0.007)	−0.032*** (0.011)		−0.036*** (0.009)	−0.035** (0.014)
Edu		−0.073 *** (0.022)	−0.076*** (0.026)		−0.058** (0.026)	−0.075*** (0.027)
Invest		0.002 (0.007)	−0.009 (0.007)		−0.001 (0.009)	−0.008 (0.008)
Temp		0.134*** (0.037)	0.080** (0.033)		0.159*** (0.048)	0.111*** (0.042)
Wind		−0.012 (0.008)	−0.003 (0.007)		−0.021** (0.009)	−0.011 (0.008)
Hum		0.006 (0.027)	0.029 (0.025)		0.002 (0.033)	0.024 (0.030)
city fixed effects	YES	YES	YES	YES	YES	YES
year fixed effects	YES	NO	YES	YES	NO	YES
*N*	2625	2625	2625	2625	2625	2625
*R*^*2*^	0.291	0.241	0.333	0.330	0.281	0.377

***p < 0.01, **p < 0.05, *p < 0.1.

*Heterogeneity test 2*: Eastern, central, and western regions. To examine the spatial heterogeneity of NTU's eco-effects, we divide the sample cities into three groups: the eastern, central, and western regions. The corresponding results are shown in Table 11. Note that the estimated coefficients of the core variable are significantly negative only in the eastern region. China is highly diversified in economic development, urbanization, and climate [39]. The degree of regional development is best in the eastern region and relatively low in the middle and western regions. Without sufficient capital and resources, the proposed NTU, an indeterminate development strategy, often fails to attract market interest. It is unsustainable in economic terms when NTU investment relies heavily on local fiscal funds or bank loans. Under tremendous pressures of economic development, the less-developed regions hesitate to give the highest priority to environmental targets. The advance of NTU potentially leads to the location of more innovative and cleaner industries in eastern city clusters and relocation of older and more dirty ones to inland/western regions [3]. Evidence shows that firms in poor areas are more likely to be more pollution-intensive, while firms in rich areas have become cleaner during the current development period [57]. This kind of regional heterogeneity naturally makes the ecological improvements from NTU problematic in less-developed middle/western regions.

**Table 11:** Heterogeneity test on the perspective of spatial location.

Variables	Eastern	Central	Western
event×time	−0.165*** (0.025)	0.015 (0.028)	0.120 (0.104)
InduStru	−0.042 (0.028)	−0.032 (0.026)	−0.036 (0.027)
Econ	−0.273*** (0.077)	−0.272*** (0.071)	−0.279*** (0.078)
Sci	−0.030** (0.012)	−0.035*** (0.012)	−0.032** (0.014)
Edu	−0.068*** (0.024)	−0.070*** (0.025)	−0.065** (0.027)
Invest	−0.008 (0.008)	−0.009 (0.007)	−0.006 (0.008)
Temp	0.100** (0.041)	0.095*** (0.033)	0.109** (0.042)
Wind	−0.007 (0.008)	−0.009 (0.008)	−0.011 (0.008)
Hum	0.026 (0.027)	0.022 (0.028)	0.018 (0.030)
city fixed effects	YES	YES	YES
year fixed effects	YES	YES	YES
*N*	1463	2359	2014
*R*^*2*^	0.345	0.372	0.395

***p < 0.01, **p < 0.05, *p < 0.1.

*Heterogeneity test 3*: Levels of PM_2.5_. We further explore the heterogeneity in the relative effects of NTU on environmental pollution across different levels of PM_2.5_. For this purpose, a quantile regression of PM_2.5_ is conducted, and the quantiles are taken to be 10%, 25%, 50%, 75%, and 90%. The baseline results are reported in Table 12. Note that for quantiles 10%, 25%, 50%, and 75%, the regression coefficients are all negative and statistically significant at the 10% level. For the 90% quantile, although the coefficient is negative, it is not significant. This endorses the argument that eco-development is not a routine issue, which has to adapt to changes [39]. The weight of the past could prove too much and severely hinder the transformation process. There is a vicious circle of environmental degradation and economic backwardness. This intertwined logic sets up serious obstacles to disentangling economic and ecological interests; this must be addressed in the NTU process.

**Table 12:** Heterogeneity test of PM_2.5_ based on the quantile regression.

Variables	Quantiles				
	10%	25%	50%	75%	90%
event×time	−0.090* (0.047)	−0.081** (0.034)	−0.071*** (0.025)	−0.057* (0.033)	−0.045 (0.052)
InduStru	0.007 (0.008)	0.020*** (0.006)	0.037*** (0.004)	0.057*** (0.006)	0.077*** (0.009)
Econ	−0.178 (0.136)	−0.270*** (0.071)	−0.383*** (0.075)	−0.525*** (0.097)	−0.663*** (0.152)
Sci	−0.025 (0.016)	−0.027** (0.011)	−0.030*** (0.008)	−0.033*** (0.011)	−0.036** (0.017)
Edu	−0.080 (0.052)	−0.082** (0.038)	−0.084*** (0.028)	−0.086** (0.037)	−0.089 (0.058)
Invest	−0.001 (0.014)	−0.001 (0.011)	−0.004 (0.008)	−0.008 (0.010)	−0.011 (0.016)
Temp	0.095 (0.098)	0.115 (0.072)	0.139*** (0.053)	0.170** (0.069)	0.199* (0.109)
Wind	0.004 (0.017)	−0.003 (0.013)	−0.012 (0.009)	−0.023** (0.012)	−0.034* (0.019)
Hum	−0.006 (0.069)	0.001 (0.051)	0.010 (0.038)	0.021 (0.049)	0.032 (0.077)
year fixed effects	YES	YES	YES	YES	YES
*N*	2625	2625	2625	2625	2625

***p < 0.01, **p < 0.05, *p < 0.1.

## 5 Conclusions and discussion

China is experiencing a dramatic and rapid transition from rural to urban society, especially after the NTU policy was issued in 2014 [3,10,18,47]. As a top-down effort to shift China's development model, NTU is simultaneously speeding up the national urbanization process and bringing about far-reaching environmental and social effects [9,14,19,24]. Continuous expansion in urban population and area is a comprehensive and systematic project, and the expected path is an intensive, intelligent, green, and low-carbon one [20,44]. Despite remarkable achievements, possibly the greatest human-resettlement experiment in history, not enough effort has been made to clearly indicate whether NTU well addresses critical ecological issues, one of its core objectives, during this unprecedented progress. Through an empirical investigation of China's NTU process, this study conducts a meticulous environmental assessment of its implementation by adopting a series of DID analyses. The quantitative results show that advancement of NTU is by and large conducive to curbing environmental pollution, using PM_2.5_ concentrations as a proxy. That is, there are positive correlations between NTU and decreased PM_2.5_ concentrations. This finding implies that NTU recognizes building up urban ecology as a high-priority target and promotes realization of green development in a comprehensive manner. Meanwhile, the ecological effect of NTU is heterogeneous in terms of city level, location, and former pollution level. The estimation results of cohort analyses show that small and medium cities benefit more than larger or central cities with respect to environmental improvements. As for spatial location, eastern cities exhibit better achievements in air quality promotion than those in western regions. The positive consequences of NTU are restricted by level of pollution. Heavier pollution adds greatly to the difficulty of abatement due to the existing economic base, industrial structure, population agglomeration, or infrastructure.

In accordance with the findings described above, this study proposes important policy recommendations and practical guidance, as follows. First, it is necessary to continue to promote NTU and to improve environmental conditions. Urbanization is a complex social system evolution, associated with a large array of changes in socioeconomic and environmental factors. Despite significant progress in urbanization, China is facing both challenges and opportunities to effectively decouple environmental emissions, such as PM_2.5_, CO_2_, and other pollutants, from socioeconomic prosperity, with the ultimate goal of sustainable development and ecological civilization [23]. NTU offers a strategic roadmap to tackle environmental problems by putting people at the center of the policy agenda [44]. However, realization of a strategy requires concrete tactics. An integrated management framework linking population, industry, space, society, resources, and climate units should be established for synergistic governance of the natural environment. Such a framework ought to endorse a win–win situation for various stakeholders through extensive coordination at both the regional and national levels. For example, all governments need to deliberately scrutinize grand construction programs as to whether they deviate from policy or alleviate rampant pollution. New evaluation systems covering more information and third-party measures are expected to comprehensively and objectively reflect both the quality of development and environmental costs.^5^ Green urbanism needs to be advocated to bring about an environmental turning point [45].

Second, it is requisite to alleviate regional disparities in a comprehensive way during implementation of NTU. There are wide variations among cities at distinct levels of the administrative hierarchy or in different regions. These variations set up obstacles to efficacious environmental governance. Tailored institutional arrangements or policies are anticipated to reduce unreasonable spatial distribution. On the one hand, the provision of public services ought to be allowed to span handily across administrative boundaries [3]. The first step is interconnection of infrastructure construction. Consequently, the fast and smooth flow of critical elements, such as human resources, information, technology, and capital, is encouraged to enter the new urbanization areas. More attention is required to focus on the attractiveness of small–medium cities in less western regions. On the other hand, local participants should rely on their own characteristics rather than national design. They need to adjust fiscal, environmental, and land use measures to local conditions. These regional characteristic NTU plans might avoid duplication of construction and timely fill the gap between changing factors and environmental outcomes. In sum, elimination of spatial mismatch and enhancement of social-spatial equality would significantly improve NTU and strengthen its ripple effects [2].

Third, it is imperative to tap innovation-driven development. The traditional method—imposing stronger regulations, shutting down polluting industries, adopting end-of-pipe treatments—represent short-term palliative measures [[56], [65], [66]]. Novel development patterns based on technological innovation and industrial structure upgrades could create a virtuous circle. Being a catalyst for new growth opportunities and competitive advantage, innovation-driven development, beyond the concentrated goals of population agglomeration and industrialization, is more likely to produce double positive externalities and be seen as an avenue to reach ecological goals in a cost-effective way. Therefore, a portfolio of far-sighted policies should be well-designed according to diverse local conditions so that proactive environmental actions (e.g., environmental innovation, eco-manufacturing, and green construction) would be triggered naturally rather than reactively throughout the urbanization process. Stricter regulation, higher pollution emissions standards, wider supervision, greener consumption style, and extended responsibilities of polluters would likely make appropriate corrections to the current paradigm and give China the level of environmental quality it is striving for [6,22,67,68]. To maximize the contribution of these policies, interventions must take into account the consistency between the characteristics of NTU and the environmental challenges it addresses.

However, the limitations of this study must be taken into consideration. While we provide NTU implementation assessment of environment by taking PM_2.5_ as a proxy, other channels exist through which NTU may impact environment. It is noted that there is a growing concern over the pollution of water [32,33] and solids [38] linked to urbanization, thus a comprehensive, dynamic indicator system of pollution is desirable. Also, residents’ health is related to environment (Wang, 2018; [17], which is also one of the connotations of a people-oriented urbanization. Future research on this topic may consider these points to seek balance between NTU and environment to achieve sustainable development.

## Author contribution statement

Jiangyan Wang; Han Lin: Conceived and designed the experiments; Wrote the paper.

Jiangyan Wang; Suwan Han: Performed the experiments; Wrote the paper.

Pingping Wu; Han Lin: Analyzed and interpreted the data; Wrote the paper.

Jingfeng Yuan: Contributed reagents, materials, analysis tools or data; Wrote the paper.

## Funding

This work was supported by the National Natural Science Foundation of China (72271126, 12271255, 71771125), Major Project of Natural Science Foundation of Jiangsu Education Department (21KJA630001, 22KJA630001), Natural Science Foundation of Jiangsu Province (BK20180820), and Qinglan Project of Jiangsu Province.

## Data availability statement

Data will be made available on request.

## Conflict of interest

The authors declare no conflict of interest.
